# Tubule-specific cyclin-dependent kinase 12 knockdown potentiates kidney injury through transcriptional elongation defects

**DOI:** 10.7150/ijbs.90872

**Published:** 2024-02-12

**Authors:** Yi-Lin Zhang, Tao-Tao Tang, Wei-Jie Ni, Zhong-Tang Li, Liang-Yun-Zi Jiang, Yao Wang, Xuan Zhou, Jing-Yuan Cao, Qing Yin, Wei Jiang, Ya-Jie Zhao, Wei-Hua Gan, Ai-Qing Zhang, Zuo-Lin Li, Yi Wen, Lin-Li Lv, Bi-Cheng Liu, Bin Wang

**Affiliations:** 1 Institute of Nephrology, Zhong Da Hospital, Southeast University School of Medicine, Nanjing, Jiangsu, China.; 2 Nanjing University of Traditional Chinese Medicine, Nanjing, Jiangsu, China.; 3 Department of Nephrology, The Affiliated Hospital of Yangzhou University, Yangzhou University, Yangzhou, Jiangsu, China.; 4 Shanghai OE Biotech Co., Ltd., Shanghai, China.; 5 Institute of Nephrology, The Affiliated Taizhou People's Hospital of Nanjing Medical University, Taizhou School of Clinical Medicine, Nanjing Medical University, Nanjing, Jinagsu, China.; 6 Department of Pediatric Nephrology, the Second Affiliated Hospital of Nanjing Medical University, Nanjing, Jiangsu, China.

**Keywords:** CDK12, Apoptosis, DNA damage, AKI, Proximal tubule, Transcriptional elongation defect, Fgf1, Cast

## Abstract

Direct tubular injury caused by several medications, especially chemotherapeutic drugs, is a common cause of AKI. Inhibition or loss of cyclin-dependent kinase 12 (CDK12) triggers a transcriptional elongation defect that results in deficiencies in DNA damage repair, producing genomic instability in a variety of cancers. Notably, 10-25% of individuals developed AKI after treatment with a CDK12 inhibitor, and the potential mechanism is not well understood. Here, we found that CDK12 was downregulated in the renal tubular epithelial cells in both patients with AKI and murine AKI models. Moreover, tubular cell-specific knockdown of CDK12 in mice enhanced cisplatin-induced AKI through promotion of genome instability, apoptosis, and proliferative inhibition, whereas CDK12 overexpression protected against AKI. Using the single molecule real-time (SMRT) platform on the kidneys of CDK12^RTEC+/-^ mice, we found that CDK12 knockdown targeted *Fgf1* and *Cast* through transcriptional elongation defects, thereby enhancing genome instability and apoptosis. Overall, these data demonstrated that CDK12 knockdown could potentiate the development of AKI by altering the transcriptional elongation defect of the *Fgf1* and *Cast* genes, and more attention should be given to patients treated with CDK12 inhibitors to prevent AKI.

## Introduction

Acute kidney injury (AKI) constitutes a medical emergency with multiple aetiologies.^1^ Medications are a common cause of AKI, especially chemotherapy-drugs^2^, which are accumulated preferentially in the renal tubular cells, leading to worsened kidney injury.^3^ Thus, elucidating the molecular mechanism and exploring interventions for drug-mediated nephrotoxicity still lie ahead.

Cyclin-dependent kinase 12 (CDK12) belongs to the cyclin-dependent kinase (CDKs) family, which was firstly named in 2001.^4^ Numerous studies have indicated that the expression of DNA damage response (DDR) genes is selectively downregulated in cancers with loss-of-function CDK12 mutations.^5^-^7^ CDK12 is thus important for maintaining genomic stability and has been extensively assessed as a potential target for cancer therapy.^8^-^11^ Dinaciclib, a novel selective CDK12 inhibitor, showed clinical efficacy in patients with cancer, such as renal cancer, myeloma, leukaemia, and lung cancer, but the incidence of AKI has been reported to reach 10-15%.^10^,^12^,^13^ However, its potential role in the pathogenesis of AKI and the corresponding genomic stability remain unclear.

Tubular epithelial cells (TECs) are the nidus of injury during AKI.^14^,^15^ CDK12 is mainly expressed in the nucleus of TEC in renal tissue.^16^ Convincing evidence has shown that CDK12 is positively correlated with renal function in patients with chronic kidney disease.^17^ Therefore, we speculated that CDK12 downregulation was involved in the progression of kidney injury. We first generated TEC-specific CDK12-knockout mice (CDK12^flox/flox^, Cdh16-Cre, CDK12^RTEC-/-^) using the Cre-LoxP system. Interestingly, we found that CDK12^RTEC-/-^ mice exhibited severe polyuria and urine retention with renal dysfunction.^18^ Such pathologic effects may result from slow transcriptional elongation dynamics of the *Slc12a1* gene.^18^ These findings suggested an intimate correlation between CDK12 loss and kidney damage. Whether TEC injury and the development of AKI are associated with the extent of CDK12 deficiency needs to be further studied.

In this study, we generated TEC-specific CDK12-knockdown mice and demonstrated that CDK12 knockdown aggravated cisplatin-induced AKI through promotion of genome instability, apoptosis, and proliferative inhibition, which is possibly regulated by transcriptional elongation defects of the *Fgf1* and *Cast* genes. Our data propose a new molecular mechanism for AKI caused by CDK12 inhibitors that could be a potential target for the prevention of AKI.

## Results

### CDK12 expression decreases in patients with AKI and multiple types of experimental AKI models

**Figure 1:**
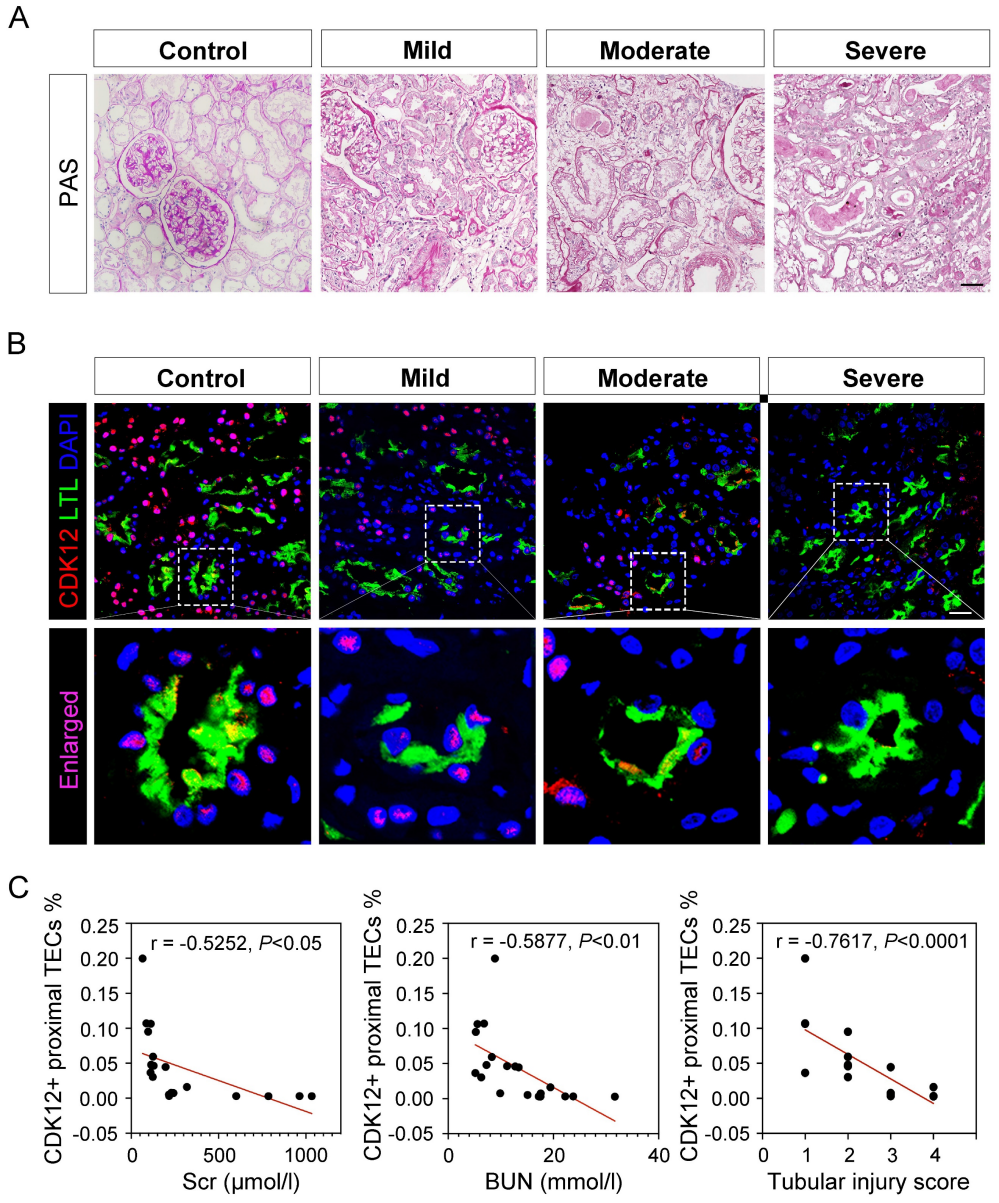
CDK12 is downregulated in kidney tissues from patients with AKI. (A) Representative micrographs show kidney morphology in different stages of AKI patients, as indicated. Nontumor kidney tissue from the patients who had renal cell carcinoma and underwent nephrectomy was used as normal controls. Images of PAS staining are shown (scale bar =50 μm). (B) Representative micrographs show CDK12 localized in LTL^+^ proximal tubules. Sequential paraffin-embedded kidney sections from patients with AKI were immunostained for CDK12 and LTL. Boxed areas are enlarged and indicate tubules of patients with AKI lack CDK12 nuclear expression (scale bar = 50 μm). (C) The correlation analysis shows an inverse correlation between the expression level of CDK12 and Scr, BUN and tubular injury score. The Spearman correlation coefficient (r) and *P* value are shown. The tubular injury scores were analysed in accordance with PAS staining. At least 20 randomly selected fields were evaluated under ×400 magnification and results were averaged for each kidney (n=20).

To test the clinical relevance of CDK12 in the pathogenesis of AKI, we conducted immunofluorescence staining in kidney biopsy specimens from 40 patients with AKI. The clinical characteristics of all patients are summarized in Supplementary Table 1. Representative images of periodic acid-Schiff (PAS) staining are presented and quantitative histological injury scoring from blinded grading of the severity of tubular injury was performed (Fig. 1A). Compared with the presence of signal in normal human kidneys, the signal for CDK12 strikingly decreased in all biopsy specimens from the patients with AKI (Fig. 1B). Notably, CDK12 was localized in the nucleus of TEC in diseased kidneys (Figure 1B, LTL^+^ proximal tubules). Furthermore, CDK12 expression in TEC correlated with tubular injury and a decline in serum creatinine (Scr) and blood urea nitrogen (BUN) (Fig. 1C).

**Figure 2:**
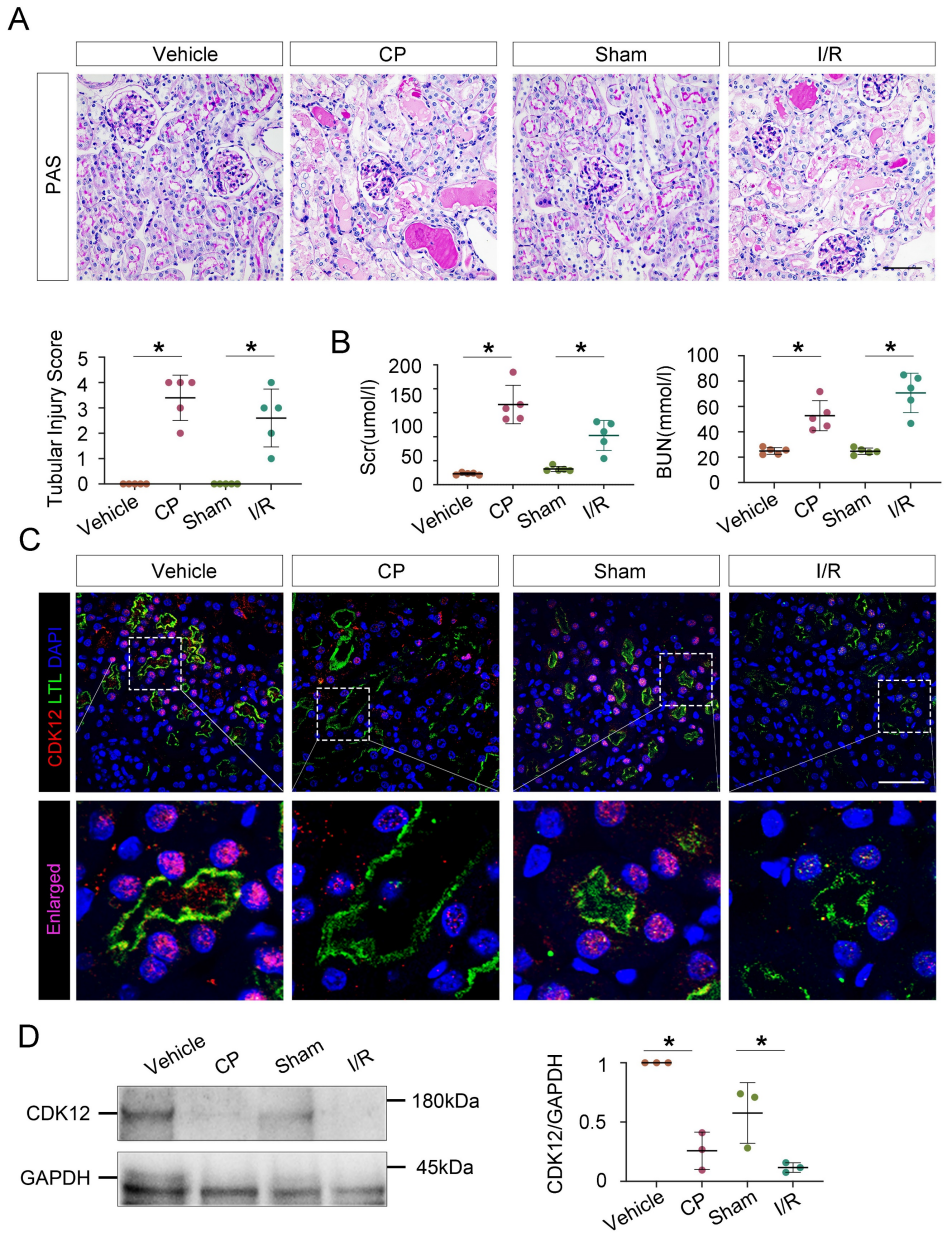
CDK12 is downregulated in cisplatin- and IR-induced AKI murine models. (A) Representative micrographs show kidney morphology in wild-type (WT) mice and mice with cisplatin- and I/R-induced AKI. Images of PAS staining are shown (scale bar =50 μm). At least 20 randomly selected fields were evaluated under ×400 magnification and results were averaged for each kidney (n=5). (B) Scr and BUN levels in the four groups, as indicated. Scr was expressed as micromoles per liter, and BUN was expressed as millimoles per liter. *P<0.05 versus WT (n=5) (C) Representative micrographs show CDK12 localized in LTL^+^ proximal tubules. Sequential paraffin-embedded kidney sections in four groups were immunostained for CDK12 and LTL. Boxed areas are enlarged and indicate tubules of mice with AKI lack CDK12 nuclear expression (scale bar = 50 μm). (D) Representative western blots showing the renal expression of CDK12 in four group. Graphical representations of CDK12 expression levels in four groups, as indicated. *P<0.05 versus WT (n=5). WT, wild type; CP, cisplatin; Scr, serum creatinine; BUN, urea nitrogen.

To further evaluate the role of CDK12 in the progression of AKI, we assessed the expression of CDK12 in different AKI models, including cisplatin- and bilateral ischemia-reperfusion (I/R)- induced AKI. Cisplatin and renal I/R robustly induced AKI in C57/Bl6 mice as indicated by morphologic injury (Fig. 2A) and marked increases in BUN, Scr and tubular injury (Fig. 2B). As shown in Figure 2, C and D, decreased CDK12 expression in the nucleus of LTL^+^ proximal tubules was obviously observed in the mice with AKI. Consistently, single-cell RNA sequencing analysis revealed that CDK12 expression was decreased in the proximal S3 segments of I/R-induced AKI mice (Supplementary Fig. 1A). Thus, our findings indicated that the decreasing expression of CDK12 is associated with the development of AKI.

### Tubular cell-specific knockdown of CDK12 aggravates cisplatin-induced AKI

**Figure 3:**
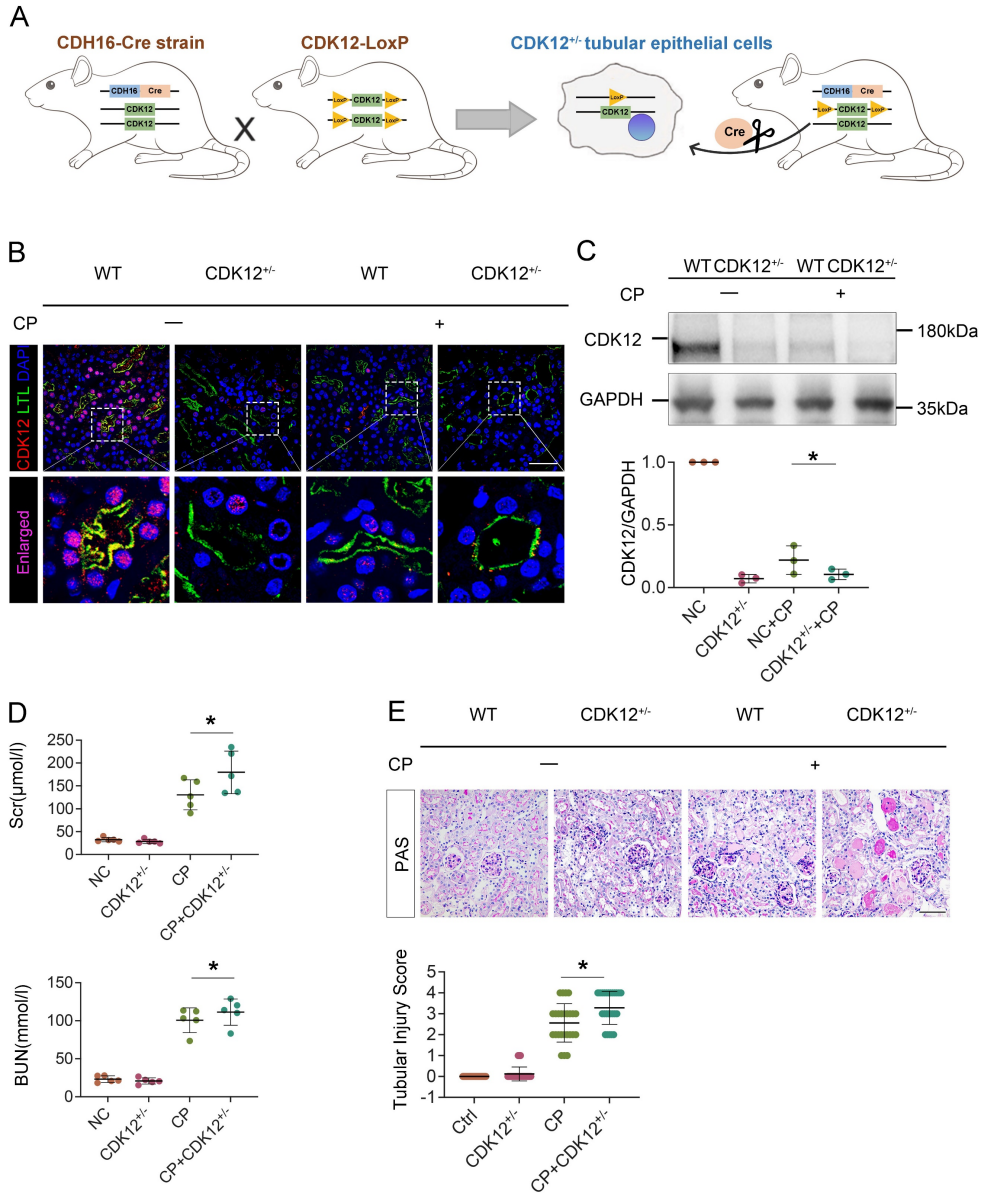
Tubular cell-specific knockdown of CDK12 facilitates cisplatin-induced AKI. (A) The mating scheme for the generation of CDK12 knockdown mice. (B) Representative immunofluorescence micrographs show CDK12 expression in WT, CDK12^RTEC+/-^ mice and the WT and CDK12^RTEC+/-^ mice injected with cisplatin. Boxed areas are enlarged and presented in the below column (scale bar = 50 μm). (C) Representative western blots showing the renal expression of CDK12 in four groups. Graphical representations of CDK12 expression levels in four groups, as indicated. *P<0.05 versus WT (n=5). (D) CDK12 knockdown increased Scr and BUN levels in CP mice. Quantitative analysis of Scr and BUN levels in four groups, as indicated. *P, 0.05 versus cisplatin alone (n=5). (E) Representative PAS micrographs show kidney morphology in four groups. Quantitative analysis of injured tubules in three groups, as indicated. Kidney sections were subjected to PAS staining. At least 20 randomly selected fields were evaluated under ×400 magnification and results were averaged for each kidney(n=5). *P, 0.05 versus cisplatin alone. CDK12^+/-^, tubular cell-specific knockdown of CDK12 mice.

To establish the role of tubular CDK12 in the kidney, we first generated TEC-specific CDK12-knockout mice (CDK12^flox/flox^, Cdh16-Cre, CDK12^RTEC-/-^) using the Cre-LoxP system and found that CDK12^RTEC-/-^ mice exhibited severe polyuria and polydipsia with mild renal dysfunction.^18^ To better mimic the downregulation of CDK12 expression via using a CDK12 inhibitor, we further generated conditional knockdown mice in which the *CDK12* gene was downregulated in TEC by using the Cre-LoxP system. Figure 3A shows the strategy for generating mice with TEC -specific knockdown of the *CDK12* gene. Mice with TEC -specific knockdown of CDK12 were named as CDK12^RTEC+/-^ mice, and age- and sex-matched CDK12-floxed wild-type littermates were considered as controls. Immunofluorescence assays showed that CDK12 was decreased in tubular epithelial cells in the mice with cisplatin-induced AKI compared with the WT mice, whereas it was further largely reduced in most of the proximal tubules in the CDK12^RTEC+/-^ mice (Fig. 3B). Western blot analysis also revealed that CDK12 expression was decreased in kidney tissue from the CDK12^RTEC+/-^ mice (Fig. 3C). We then detected the role of CDK12 knockdown in cisplatin-induced AKI. As shown in Figure 3, D and E, we did not detect a renal functional decline in the CDK12 knockdown mice. However, the levels of Scr and BUN were further increased by *in vivo* CDK12 knockdown after cisplatin treatment (Fig. 3D). Moreover, PAS staining demonstrated that *in vivo* CDK12 knockdown significantly exacerbated morphologic tubular injury after cisplatin treatment. This injury was characterized by loss of the brush border, tubular lumen enlargement and TEC loss (Fig. 3E). Taking together, these data indicated that knockdown of CDK12 in TECs accelerates kidney damage and functional decline.

### Knockdown of CDK12 expression *in vivo* and *in vitro* aggravates cisplatin-induced DNA damage

**Figure 4:**
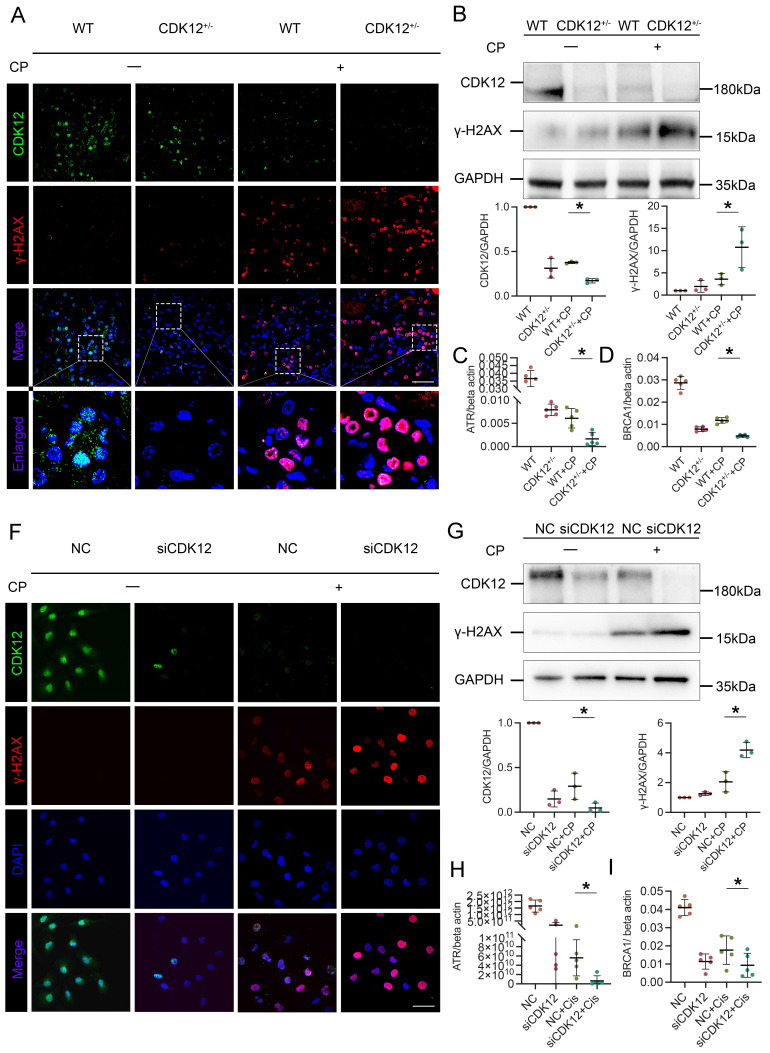
CDK12 knockdown aggravates cisplatin-induced tubular cell DDR both *in vivo* and *in vitro*. (A) Representative immunostaining micrographs show CDK12 (green) and γ-H2AX (red) expression in different *in vivo* groups. Boxed areas are enlarged and presented in the below column (scale bar = 50 μm). (B) Representative western blots showing the renal expression of CDK12 and γ-H2AX in four groups. Graphical representations of CDK12 and γ-H2AX expression levels in four groups, as indicated. *P<0.05 versus cisplatin alone (n=5). (C) Expression of ATR and (D) BRCA1 in different groups was assessed by real-time PCR. *P<0.05 versus cisplatin alone (n=5). (F) Representative immunostaining micrographs show CDK12 (green) and γ-H2AX (red) expression in different *in vitro* groups. Boxed areas are enlarged and presented in the below column (scale bar = 50 μm). (G) Representative western blots showing the HK-2 expression of CDK12 and γ-H2AX in four groups. Graphical representations of CDK12 and γ-H2AX expression levels in four groups, as indicated. *P<0.05 versus cisplatin alone (n=3). (H) Expression of ATR and (I) BRCA1 in different groups was assessed by real-time PCR. *P<0.05 versus cisplatin alone (n=3).

To identify the underlying mechanism of CDK12 in AKI, we first performed total RNA-seq to characterize the transcriptome signature of the CDK12 knockout kidneys (Supplementary Fig. 1B). GO analysis showed that the genes differentially expressed (DE) following CDK12 knockout shared some common biological process terms, such as response to DNA damage and apoptotic response. We further investigated the effects of CDK12 knockdown on DNA damage after cisplatin treatment. Double immunofluorescence assays and western blot analysis indicated that cisplatin treatment induced the expression of gamma histone variant (γ-H2AX), a strong marker of DNA double-strand breaks (DSBs) as an early response to DNA damage in cisplatin-induced AKI.^19^ The expression of γ-H2AX was further increased by *in vivo* CDK12 knockdown after cisplatin treatment (Fig. 4A). Western blot analysis confirmed these results (Fig. 4B). Real-time PCR results showed that the levels of ATR and BRCA1, central DNA repair genes^20^, were significantly decreased in the CDK12^RTEC+/-^ mice after cisplatin injection (*P* < 0.05; Fig. 4C, D).

We further examined the DNA damage *in vitro*. We first transfected a cultured tubular epithelial cell line (HK-2) with CDK12 siRNA to knockdown its expression. Then we treated these cells with cisplatin for 24 h. The immunofluorescence and western blot results indicated that the expression of γ-H2AX in nuclear foci was significantly increased in the siCDK12 cells (Fig. 4F, G). Real-time PCR results indicated that the knockdown of CDK12 expression significantly blocked the expression of ATR and BRCA1 after cisplatin treatment (*P* < 0.05; Fig. 4H, I). These data suggested the important role of CDK12 in cisplatin-induced DNA damage and cellular injury.

### Knockdown of CDK12 expression *in vivo* and *in vitro* aggravates cisplatin-induced tubular cell apoptosis

**Figure 5:**
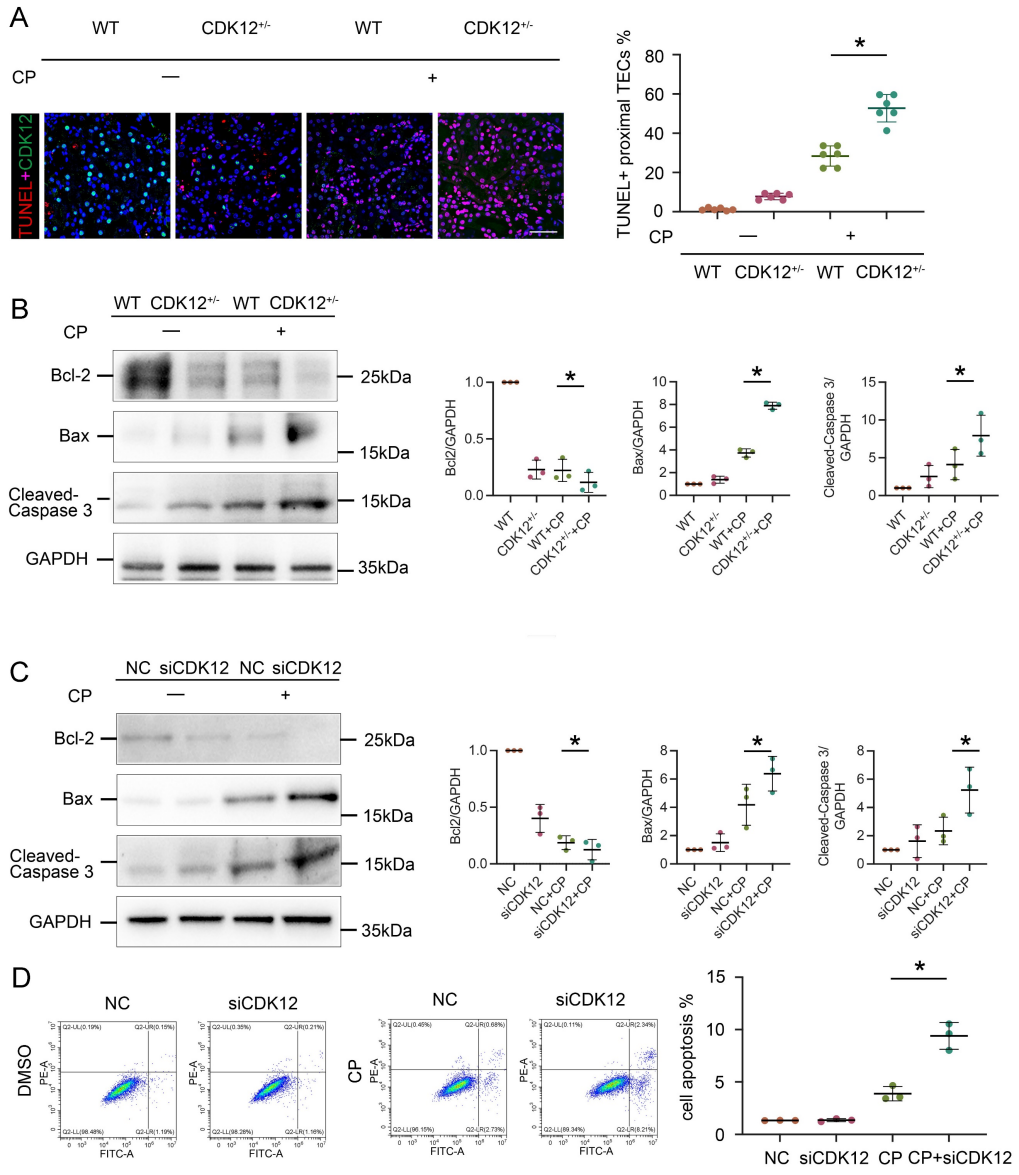
CDK12 knockdown aggravates cisplatin-induced tubular cell apoptosis both *in vivo* and *in vitro*. (A) Representative immunostaining micrographs show CDK12 (green) and TUNEL (red) expression in different *in vivo* groups (scale bar = 50 μm). Quantitative analysis of TUNEL^+^ proximal tubules in four groups, as indicated. At least 20 randomly selected fields were evaluated under ×400 magnification and results were averaged for each kidney. *P, 0.05 versus cisplatin alone (n=5). (B) Representative western blots showing the renal expression of Bcl-2, Bax and cleaved-Caspase3 in four groups. Graphical representations of Bcl-2, Bax and cleaved-Caspase3 levels in four groups, as indicated. *P<0.05 versus cisplatin alone (n=5). (C) Representative western blots showing the HK-2 expression of Bcl-2, Bax and cleaved-Caspase3 in four groups. Graphical representations of Bcl-2, Bax and cleaved-Caspase3 levels in four groups, as indicated. *P<0.05 versus cisplatin alone (n=3). (D) Annexin V-FITC/PI double staining and a flow cytometer for apoptotic cells in four groups, as indicated. Quantitative analysis of apoptotic proximal tubules in four groups. *P, 0.05 versus cisplatin alone (n=3).

We next investigated the relevance of CDK12 knockdown in apoptosis. As shown in Figure 5A, representative terminal deoxynucleotidyl transferase-mediated dUTP nick end labelling (TUNEL) staining revealed that TUNEL-positive TECs were significantly increased in the kidneys of the CDK12^RTEC+/-^ mice after cisplatin injection. We further examined the expression of the apoptosis-related proteins Bcl-2, Bax and cleaved caspase 3. Members of the Bcl-2 family are important regulators of the mitoptotic pathways, among which proapoptotic Bcl2 associated X (Bax) and antiapoptotic Bcl-2 are mainly located in the mitochondrial membrane and regulate the activation of caspase-3, finally inducing apoptosis.^21^ Western blot analysis showed that cisplatin markedly inhibited the expression of Bcl-2 and induced the expression of Bax and cleaved caspase-3, whereas in the CDK12^RTEC+/-^ mice after cisplatin treatment, this process was exacerbated (Fig. 5B).

We then examined the effect on apoptosis of siRNA-mediated knockdown of CDK12 in the cisplatin-treated tubular cell line. Consistently, CDK12 knockdown markedly increased the expression of Bax and decreased the expression of cleaved caspase-3 and Bcl-2 in the cisplatin-treated HK-2 cells (Fig. 5C). This observation was further verified in the detection of apoptotic cells by Annexin V-FITC/PI double staining and a flow cytometer (Fig. 5D). Taken together, these results suggest that CDK12 knockdown induces tubular apoptosis both *in vivo* and* in vitro*.

### CDK12 knockdown abrogates tubular cell proliferation after cisplatin treatment

**Figure 6:**
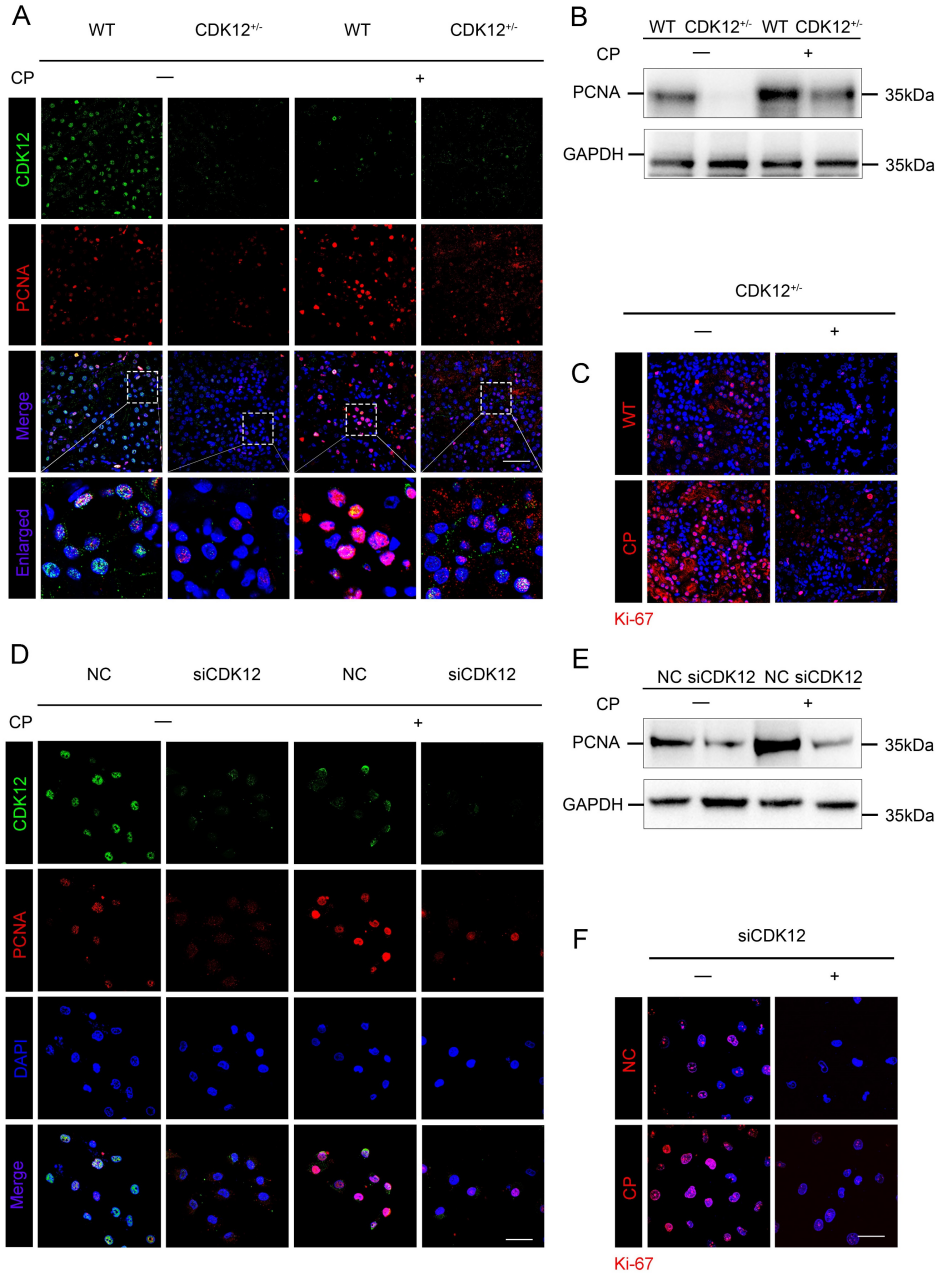
CDK12 knockdown abrogates tubular cell proliferation after cisplatin treatment both *in vivo* and *in vitro*. (A) Representative immunostaining micrographs show CDK12 (green) and PCNA (red) expression in different *in vivo* groups. Boxed areas are enlarged and presented in the below column (scale bar = 50 μm). (B) Representative western blots showing the renal expression of PCNA in four groups, as indicated. (C) Representative immunostaining micrographs show Ki-67 expression in four groups (scale bar = 50 μm). (D) Representative immunostaining micrographs show CDK12 (green) and PCNA (red) expression in different *in vitro* groups (scale bar = 50 μm). (E) Representative western blots showing the HK-2 expression of PCNA in four groups, as indicated. (F) Representative immunostaining micrographs show Ki-67 expression in four groups (scale bar = 50 μm).

We next examined the role of CDK12 in proliferation. As shown in Figure 6 A-C, cisplatin administration significantly increased the expression of the proliferation-related genes proliferating cell nuclear antigen (PCNA) and Ki-67, whereas knockdown of CDK12 significantly attenuated cisplatin-induced proliferation. Consistently, CDK12 knockdown markedly reduced cisplatin-induced HK-2 cell proliferation *in vitro* (Fig. 6 D-F). Our data indicate that CDK12 knockdown induced proliferation inhibition both *in vivo* and* in vitro*.

### Restoring CDK12 rescues cisplatin-induced tubular injury

**Figure 7:**
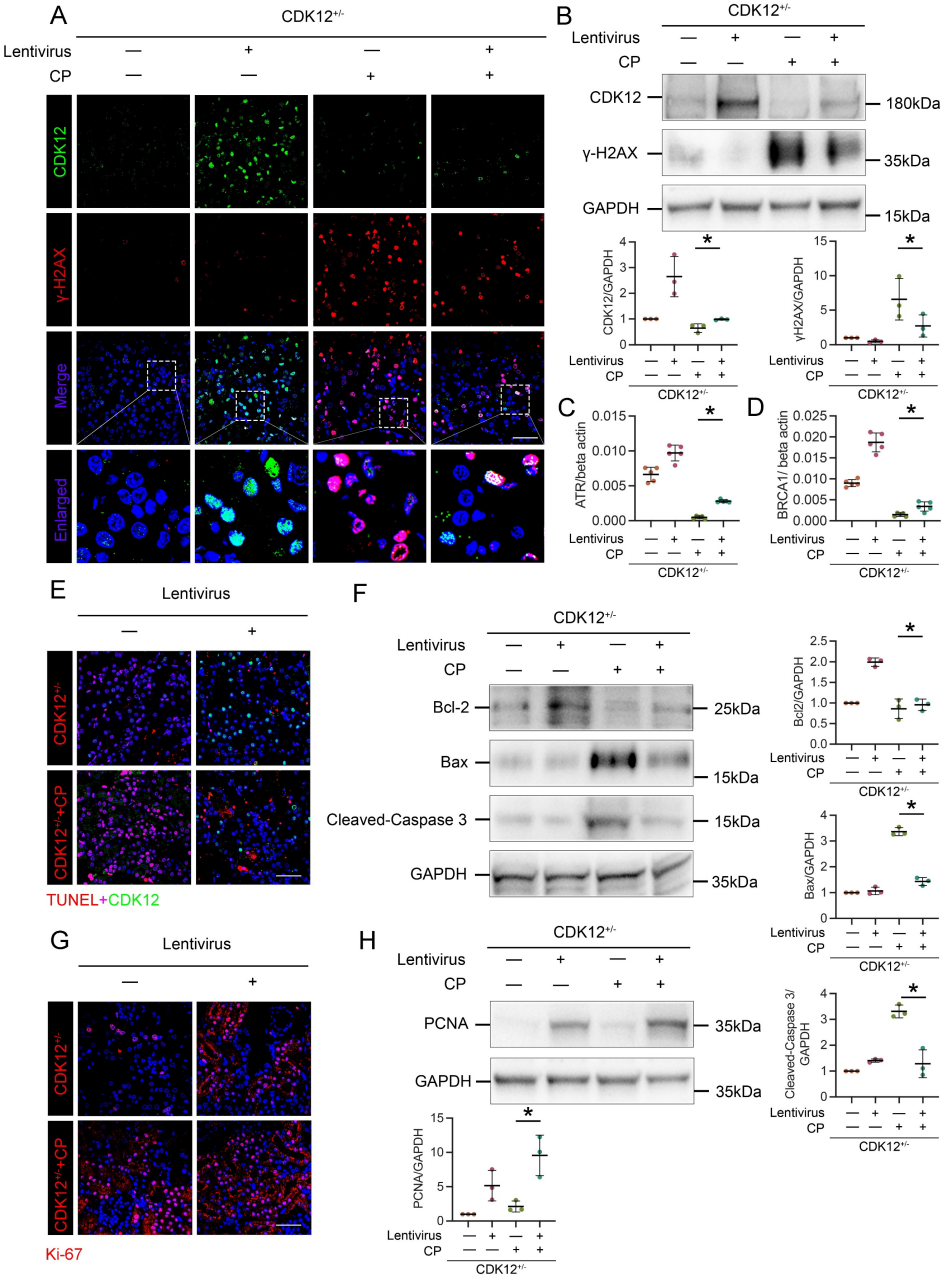
Overexpression of CDK12 improves tubular injury after cisplatin treatment *in vivo*. (A) Representative immunostaining micrographs show CDK12 (green) and γ-H2AX (red) expression in *in vivo* four groups, as indicated. Boxed areas are enlarged and presented in the below column (scale bar = 50 μm). (B) Representative western blots show that γ-H2AX expression was downregulated after injection of GFP-labelled CDK12 overexpression lentivirus. Graphical representations of CDK12 and γ-H2AX levels in four groups, as indicated. *P<0.05 versus CDK12^RTEC+/-^ with cisplatin (n=5). (C) Expression of ATR and (D) BRCA1 in different groups was assessed by real-time PCR. *P<0.05 versus CDK12^RTEC+/-^ with cisplatin (n=5). (E) Representative immunostaining micrographs show CDK12 (green) and TUNEL (red) expression in different groups (scale bar = 50 μm). (F) Representative western blots showing the renal expression of Bcl-2, Bax and cleaved-Caspase3 in four groups. Graphical representations of Bcl-2, Bax and cleaved-Caspase3 levels in four groups, as indicated. *P<0.05 versus CDK12^RTEC+/-^ with cisplatin (n=5). (G) Representative immunostaining micrographs show Ki-67 expression in four groups (scale bar = 50 μm). (H) Representative western blots showing the renal expression of PCNA in four groups. Graphical representations of PCNA levels in four groups, as indicated. *P<0.05 versus CDK12^RTEC+/-^ with cisplatin (n=5).

To further confirm the role of CDK12 in kidney damage, we administered GFP-labelled CDK12 overexpression lentivirus to 4-week-old CDK12^CTRL^ and CDK12^RTEC+/-^ mice. As shown in Figure 7 A and B, CDK12 was significantly induced in kidneys after injection of CDK12 overexpression lentivirus. The cisplatin-induced DNA damage was also significantly blocked by overexpression of CDK12 (Fig. 7 A-D). In addition, the immunofluorescence and western blot showed that CDK12 overexpression reduced cisplatin- and knockdown of CDK12- induced apoptosis and proliferative inhibition (Fig. 7 E-H). Consistently, CDK12 overexpression also significantly attenuated the extent of cellular apoptosis and DNA damage in cultured HK-2 cells induced by cisplatin (Supplementary Fig. 3).

### The influence of CDK12 knockdown on transcription elongation

**Figure 8:**
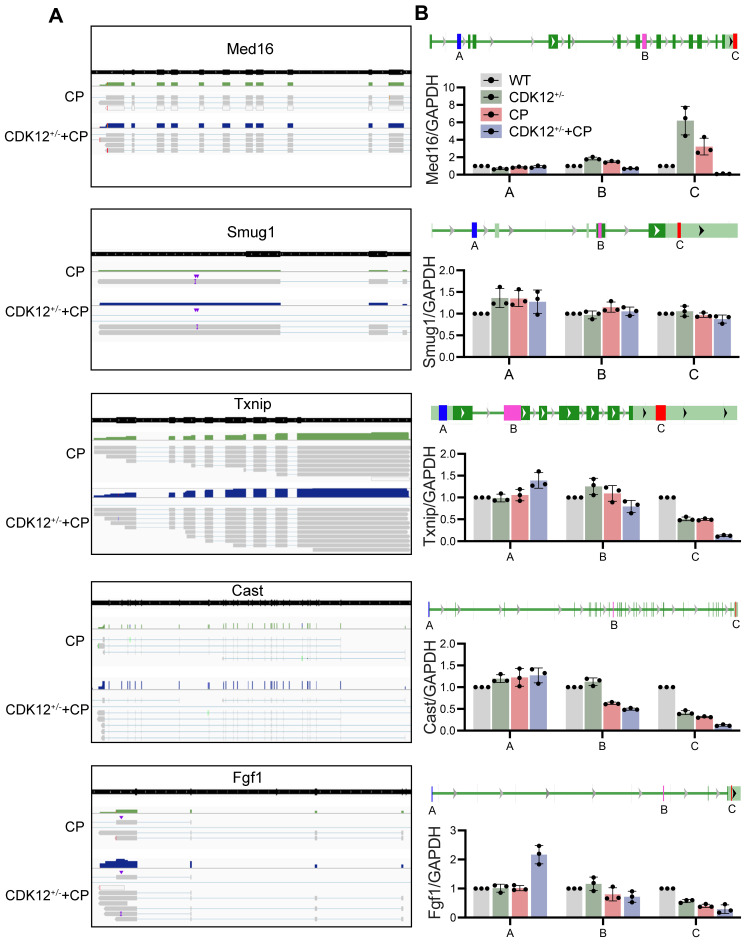
SMRT sequencing reveals transcriptional elongation defects following CDK12 knockdown during AKI. (A) Integrative Genomics Viewer (IGV) representation shows the mapping of SMRT sequencing reads at the *Med16*, *Smug1*, *Txnip*, *Cast*, and *Fgf1* genes. The presence of elongation defect due to CDK12 knockdown in mice treated with cisplatin as in number of short transcrips. (B) Three primers (located near the 5′ untranslated regions, coding sequence, 3′ untranslated regions) are labeled in blue, pink and red, respectively. Expression of Med16, Smug1, Txnip, Cast, and Fgf1 at selected regions in different groups was assessed by real-time PCR. Error bars indicate mean values of ±SD, n=3.

**Figure 9:**
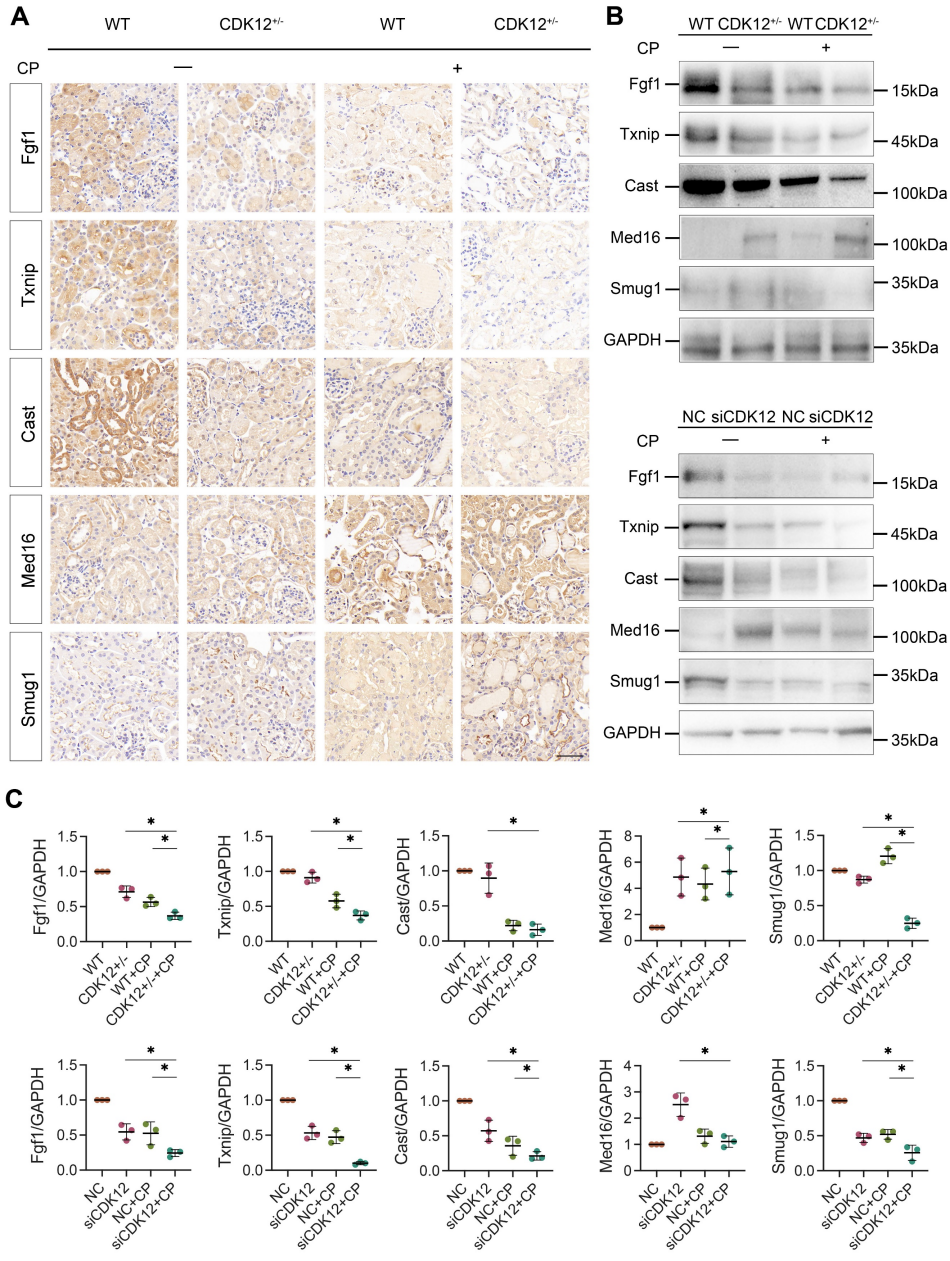
(A) Representative immunostaining micrographs show Med16, Smug1, Txnip, Cast, and Fgf1 expression in different groups, as indicated. (scale bar = 50 μm). (B) Representative western blots showing the expression of Med16, Smug1, Txnip, Cast, and Fgf1 in different groups both *in vitro* and* in vivo*. (C) Graphical representations of Med16, Smug1, Txnip, Cast, and Fgf1 levels in different groups, as indicated. *P<0.05 versus cisplatin alone or CDK12 knockdown with cisplatin (n=5).

To further clarify whether CDK12 knockdown affected the expression of genes involved in the DDR and apoptosis, we analysed the gene expression profiles in a cisplatin-induced AKI murine model treated with and without CDK12 knockdown. The main GO categories for differentially expressed genes were focused on DNA damage, metabolic process and apoptosis (Supplementary Fig. 2 A-B). To further elucidate the transcriptional effect of CDK12 knockdown on genes in these terms, we performed a long-read sequencing platform-single-molecule real-time sequencing (SMRT) in cisplatin-induced AKI murine model treated with and without CDK12 knockdown, to identify the formation of novel transcripts involved in DNA damage and apoptosis genes. The data showed that the *Med16*, *Smug1*, and *Txnip* genes involved in DDR and the *Cast* and *Fgf1* genes involved in apoptosis have short transcript variants, indicating a possible elongation defect (Fig. 8A). To pursue this hypothesis, we designed three primers (located near the 5′ untranslated regions, coding sequence, 3′ untranslated regions) along these five genes (Fig. 8B). Interestingly, we found a gradual decline in expression from the 5′ to the 3′ end of the *Cast*, *Fgf1*, and *Txnip* genes following CDK12 knockdown after cisplatin treatment (Fig. 8B). Immunohistochemistry and western blot results revealed that CDK12 knockdown repressed the expression of Cast, Fgf1, and Txnip both *in vivo* and* in vitro* (Fig. 9). Conversely, overexpression of CDK12 after cisplatin treatment largely preserved the expression of Cast, Fgf1, and Txnip in tubules (Supplementary Fig. 4 A). However, we did not detect consistent expression of the *Med16* and *Smug1* genes, indicating that CDK12 knockdown did not correlate with the formation of novel transcripts of the *Med16* and *Smug1* genes. These data suggest the important role of CDK12 in transcriptional elongation of the *Cast*, *Fgf1*, and *Txnip* genes in cisplatin-induced AKI.

### CDK12 knock-down aggravates cisplatin-induced DNA damage and apoptosis by reducing Cast and Fgf1

**Figure 10:**
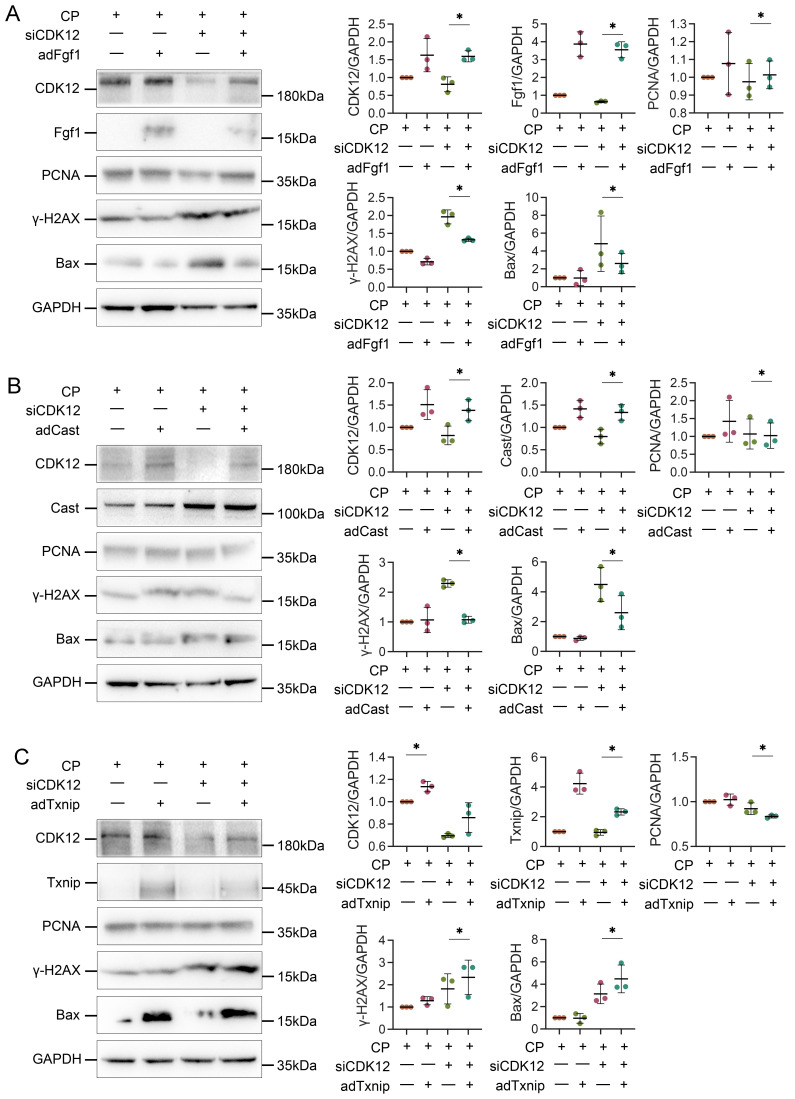
Overexpression of Txnip, Cast and Fgf1 affects apoptosis, DDR and proliferation. (A) Representative western blots show Fgf1 overexpression inhibits siCDK12-induced γ-H2AX and Bax expression and promotes PCNA expression. Graphical representations of protein expression levels in four groups, as indicated. *P<0.05 versus siCDK12 with cisplatin (n=3). (B) Representative western blots show Cast overexpression inhibits siCDK12-induced Bax expression. Graphical representations of protein expression levels in four groups, as indicated. *P<0.05 versus siCDK12 with cisplatin (n=3). (C) Representative western blots showing the expression of Txnip, PCNA, γ-H2AX and Bax in different groups. Graphical representations of protein expression levels in four groups, as indicated. *P<0.05 versus siCDK12 with cisplatin (n=3). ptFgf1, Fgf1 overexpression plasmid; ptCast, Cast overexpression plasmid; ptTxnip, Txnip overexpression plasmid.

We next examined the potential mechanism by which CDK12 knockdown aggravates cisplatin-induced DNA damage and apoptosis. We transfected HK-2 cells with Cast, Fgf1, and Txnip expression plasmids. As shown in Figure 10, A and B, Cast and Fgf1 overexpression significantly blocked the siCDK12-induced expression of Bax. Moreover, Fgf1 overexpression repressed the expression of γ-H2AX and enhanced the expression of PCNA. These findings suggest that downregulation of Cast and Fgf1 could induce DNA damage and apoptosis. Unexpectedly, overexpression of Txnip aggravated rather than attenuated kidney injury via DNA damage and apoptosis (Fig. 10C), which suggested that aggravation of kidney injury by CDK12 knockdown may not act through downregulation of Txnip. These data clearly demonstrated that CDK12 knockdown induces transcriptional elongation defects in *Cast* and *Fgf1* cells, aggravating cisplatin-induced DNA damage and apoptosis.

## Discussion

In this study, we found that CDK12 downregulation is involved in AKI. Knockdown of CDK12 leads to aggravated kidney injury, as shown in CDK12^RTEC+/-^ mice, accompanied by DNA damage, apoptosis and proliferative inhibition. Importantly, CDK12 knockdown reduced the elongation rates of the genes *Fgf1* and *Cast*, which are related to DDR and apoptosis. Hence, our findings suggest a critical role for CDK12 knockdown in AKI, which may provide a new preventive strategy for the prevention or treatment of AKI.

Recently, the incidence of drug-induced AKI has increased remarkably. Prospective cohort studies of AKI have shown that the frequency of drug-induced AKI is nearly 14-26% in adults.^22^,^23^ Of note, chemotherapy-drugs are a common cause of AKI due to their accumulation in the renal tubular cells, which subsequently causes the tubular cell injury.^24^,^25^ Previous studies have demonstrated that CDK12 regulates the early termination of POLII elongation and intronic polyadenylation in a gene-selective manner, especially controlling the expression of long genes such as BRCA1 and ATR involved in the DNA damage response.^7^,^26^,^27^ It has been confirmed that CDK13, also as components of the positive transcription elongation factor b (P-TEFb) complex, is involved in renal injury, while the direct association between CDK12 and renal injury remains largely unknown^37^. In 2016, Zhang et al. first designed a CDK12/CDK13 inhibitor, THZ531, which treated leukaemia cells by inducing a strong DNA damage response (DDR).^8^ After that, increasing evidence from trials of CDK12 inhibitors suggested that CDK12 was a potential target for a variety of cancers.^28^,^29^ Notably, 10% of the individuals developed AKI in these studies, and the potential mechanism is not well understood. Previously, we found that CDK12 knockout in TECs caused urine concentration defects due to slow *Slc12a1* gene transcriptional elongation dynamics and observed that CDK12 regulated DDR in AKI.^18^,^30^ However, it is unclear whether CDK12 knockdown can aggravate kidney injury through transcriptional elongation of genes involved in the DDR.

The DDR signaling cascade is pivotal for postinjury repair after AKI. Emerging evidence suggests that DDR-induced genomic instability plays an important role in the progression of AKI to CKD.^31^ Defective DDR not only leads to kidney injury, but also induces the expression of some proapoptotic proteins and cell cycle inhibitors.^32^ Thus, understanding the mechanism that triggers DDR is of great importance. In the present study, we found that mice with TEC-specific CDK12-knockdown could cause an early response to DNA damage and apoptosis in cisplatin-treated tubular cells as well as tubular cells proliferative inhibition. More importantly, CDK12 rescue could restore all of the abovementioned changes. Encouragingly, CDK12 expression was also decreased in patients with AKI, predominantly in tubular epithelial cells, which was negatively correlated with increases in Scr, BUN and tubular injury scores. In fact, the CDK12 activity is regulated by factors such as cell cycle proteins, post-translational modifications, and transcription factors.^38^ Previous studies have shown that cell cycle arrest and acetylation and lactylation modifications occur after injury in several models of AKI.^39^, ^40^ Clearly, these data showed that CDK12 knockdown in tubular cells could induce cellular injury through DNA damage and subsequently cause apoptosis and inhibition of proliferation.

Notably, CDK12 depletion does not change transcription globally, but alters a subset of long genes (>45 kb). As we know, DDR genes are preferentially sensitive to CDK12 deletion due to their relatively longer lengths.^7^ The other interesting observation in this study is that CDK12 knockdown caused transcriptional elongation defects of the DDR and apoptosis genes,* Fgf1 Cast* and *Txnip.* The Fgf1 C-terminal domain inhibits p53-dependent apoptosis by decreasing p53 phosphorylation on serine 15.^33^ In addition to apoptosis, Fgf1 is also involved in the regulation of the DDR. Several studies have found that Fgf1 induces DNA damage sensing and causes DSBs repair by focal recruitment of the ssDNA protective protein RPA and associated phosphorylation of ATM, which leads to the kinetics of high-fidelity HR DNA damage repair activation.^34^ Supporting this notion, we found that overexpression of Fgf1 improved DNA damage by CDK12 knockdown *in vitro*. Moreover, cleavage by calpain (specifically inhibited by cast) generates a potent proapoptotic 18-kDa fragment that promotes apoptotic cell death.^35^ Additionally, overexpression of Txnip *in vitro* aggravated CDK12 knockdown-induced tubular cell DDR and apoptosis. Supporting our findings, it was previously reported that Txnip promoted the synthesis of reactive oxygen species (ROS) by directly binding to Trx, leading to the activation of apoptosis and the accumulation of DNA damage.^36^ Thus, we believe that CDK12 knockdown-induced renal tubular cell apoptosis and DNA damage was mainly mediated by the downregulation of Fgf1 and Cast.

In conclusion, this study demonstrated that downregulation of CDK12 gene expression might be involved in the development of AKI by inducing genome instability, apoptosis, and proliferation inhibition through transcriptional elongation defects of *Fgf1* and *Cas*t. It is therefore indicated that targeted activation of tubular CDK12 could be a plausible strategy for therapeutic intervention of AKI.

## STAR Methods

### Mice

All animal studies conducted were approved by the ethics committees for animal experimentation of Southeast University. CDK12^RTEC+/-^ mice (CDK12^flox/flox^, Cdh16-Cre; weighing approximately 20-22 g) on a C57BL/6 background were provided by Dr. Yao Wang (Department of Nephrology, The Affiliated Hospital of Yangzhou University, Yangzhou University, Yangzhou, Jiangsu, China). *In vivo* virus transduction to overexpress CDK12 was performed as described previously with slight modifications.^18^ Briefly, 50 μl of filter-purified lentivirus cocktail (EGFP or EGFP-CDK12, ∼2×10^6^ IU/μl) was injected into each kidney. Age and weight-matched C57BL/6 (wild-type) mice were purchased from Vital River Laboratory Animal Technology Co., Ltd. (Beijing, China). AKI was induced in mice by a single intraperitoneal injection of 18 mg/kg cisplatin (Sigma), while the control groups were injected with saline only. Mice were sacrificed 2 days after administration, and kidney tissue and blood were collected for further studies. Serum creatinine and urea nitrogen were measured using the serum creatinine and urea nitrogen assay kits (S03076, Leinuo) at Servicebio Technology Co., Ltd (Wuhan, China).

### Morphological studies and tubular injury scoring

Formalin-fixed, paraffin-embedded mouse kidney sections (4-μm thickness) were prepared by a routine procedure. Periodic acid-Schiff (PAS) staining was conducted by a standard protocol. A semiquantitative score of renal tubular injury was performed according to the following methods: 0, no injury; 1, <25%; 2, 25~50%; 3, 50~75%; 4, >75%. The average score of 5 random sections was calculated as the tubular injury score. Immunohistochemical staining was performed using a routine protocol. The antibodies used were as follows: anti-Med16 (ab130996; Abcam), anti-Smug1 (ab192240; Abcam), anti-Txnip (ab188865; Abcam), anti-Cast (ab226249; Abcam), and anti-Fgf1 (ab207321; Abcam). Human biopsy sequential sections were obtained from the Institute of Nephrology, Zhong Da Hospital. All studies involving human kidney sections were approved by the Institutional Ethics Committee at the Zhong Da Hospital, Southeast University School of Medicine.

### Cell culture, transfection, and cell treatment

The human renal tubular epithelial cell line HK-2 was obtained from the American Type Culture Collection (Manassas, VA) and cultured in DMEM/F12 with 10% FBS in a 37 °C incubator with 5% CO2. Cells were pretreated with cisplatin (5 μg/ml) for 24 h. CDK12 siRNA and negative control (NC) were designed and synthesized by Hanbio (Shanghai, China). The sequences of CDK12 siRNA and NC were 5'-GCCAGCAUUUAGUCAGGUUTT-3' and 5'-UUCUCCGAACGUGUCACGUTT-3', respectively. Cast, Txnip, and Fgf1 expression plasmids and a negative control (NC) were designed and synthesized by GenScript (Nanjing, China). HK-2 cells were transfected with siRNA and plasmids using Lipofectamine 3000 (Invitrogen, Grand Island, NY) according to the manufacturer's instructions. Whole-cell lysates were prepared and subjected to western blot analyses. Some cells were also detected by immunofluorescence and flow cytometry analysis.

### Western blot analysis

Nearly 15 mg of total kidney tissue was homogenized in SDS lysis buffer, sonicated, and heated at 95 °C. Lysates were cleared by centrifuging (15,000×g at 4°C for 15 min). 15 μL of total lysate was loaded onto 11% SDS-PAGE and subjected to electrophoresis (140 V, room temperature). Proteins were transferred onto PVDF membranes at 100 V on ice for 1 h. Membranes were incubated in 5% bovine standard solution (BSA) prepared in Tris-buffered saline containing Tween-20 (TBST) for 1 h at room temperature on an orbital rocker. Membranes were probed with: anti-γH2AX (dilution 1:1000; ab26350; Abcam), anti-CDK12 (dilution 1:1000; GTX130809; GeneTex), anti-Bcl-2 (dilution 1:1000; 3498, Cell Signaling Technology), anti-Bax (dilution 1:1000; 89477, Cell Signaling Technology), anti-cleaved caspase-3 (dilution 1:500; ab13847; Abcam), anti-PCNA (dilution 1:1000; ab29; Abcam), anti-Med16 (dilution 1:1000; ab130996; Abcam), anti-Smug1 (dilution 1:1000; ab192240; Abcam), anti-Txnip (dilution 1:1000; ab188865; Abcam), anti-Cast (dilution 1:1000; ab226249; Abcam), and anti-Fgf1 (dilution 1:1000; ab207321; Abcam). After primary antibody incubation blots were washed three times with TBST, HRP-conjugated secondary antibodies (#7074, CST, dilution 1:2000) were probed for 1 h at room temperature prepared in TBST. Finally, blots were washed with TBST for 5 min each at room temperature. After applying the ECL color reagent and performing dark chamber exposure imaging, the gray value of the images was analyzed using ImageJ software v1.8.0 (NIH) and The final relative quantification values are the ratio of net band to net loading control. GraphPad Prism 9 was used to obtain statistical figures.

### Immunofluorescence staining

Immunofluorescence analysis was performed on kidney sections (2-μm thickness) and HK-2 cells. Slides were incubated with antibodies against anti-γH2AX (ab26350, Abcam), anti-CDK12 (ab246887, Abcam), anti-Ki-67 (ab15580, Abcam), anti-PCNA (ab29, Abcam), and LTL (FL-1321-2, Vector Laboratories) and incubated with secondary antibodies (ab150114 and ab150077, Abcam). Nuclei were stained with DAPI (Sigma-Aldrich) according to the manufacturer's instructions. Under a confocal microscope (FV3000, Olympus), 10 fields of view were randomly assigned, and the number of CDK12-positive cells was counted in a blinded manner.

### RT and real-time PCR

A TRIzol RNA isolation system (TaKaRa) was used to extract total RNA from kidney tissue and HK-2 cells. Complementary DNA was synthesized using a HiScript III RT SuperMix kit (R323, Vazyme). Real-time PCR was performed using the ChamQ SYBR qPCR Master Mix kit (Q341, Vazyme). The sequences of the primer pairs are shown in Supplementary Table 2. PCR data were calculated using the 2^-ΔΔCT^ formula.

### TUNEL staining assay and flow cytometry analysis of cell apoptosis

Cell apoptosis was assessed by both TUNEL staining and flow cytometry analysis. Paraffin sections (2-μm thickness) and HK-2 cells were fixed and stained for TUNEL staining with a TUNEL staining kit (C1090, Beyotime) according to the manufacturer's instructions. Flow cytometry analysis of apoptotic cells was performed using the Annexin V-FITC apoptosis detection kit (KGA108, KeyGEN BioTECH).

### RNA sequencing and enrichment analyses

Genomic RNA was isolated from the kidney cortex of the CDK12^RTEC+/-^ mice treated with saline or cisplatin. RNA concentrations were measured using a NanoDrop 2000 spectrophotometer (Thermo Scientific, USA). Total RNA integrity was assessed using the Agilent 2100 Bioanalyzer (Agilent Technologies, Santa Clara, CA, USA). Then, RNA sample libraries were prepared using a VAHTS Universal V6 RNA-seq Library Prep Kit according to the manufacturer's instructions. Transcriptome sequencing and analysis were conducted by OE Biotech Co., Ltd. (Shanghai, China). Briefly, the libraries were sequenced on an Illumina NovaSeq 6000 platform, and 150 bp paired-end reads were generated. The raw sequencing reads were first processed for FASTQ conversion and demultiplexing. The clean reads were mapped to the reference genome using HISAT. The FPKM of each gene was calculated, and the read counts of each gene were obtained by HTSeq-count. PCA was performed using R (v 3.2.0) to evaluate the biological duplication of samples. Differentially expressed genes (DEGs) were determined via DESeq2. Significant DEGs were defined as those with >1.5-fold or <0.7-fold change. Based on the hypergeometric distribution, GO and KEGG pathway enrichment analyses of DEGs were performed to screen the significantly enriched terms using R (v 3.2.0).

### Single molecule real-time (SMRT) sequencing

Genomic DNA was isolated from the kidney cortex of CDK12^RTEC+/-^ mice using a SMARTer PCR cDNA Synthesis Kit (Clontech, USA) according to the manufacturer's protocol. Library preparation and sequencing were conducted by OE Biotech Co., Ltd., (Shanghai, China) using SMRTbellTM Template Prep Kit 1.0-SPv3, Sequel Binding and Internal Ctrl Kit 3.0, Sequel Sequencing Kit 3.0 and SMRT Cell 1 M v3 LR Tray (Pacific BioSciences, Melon Park, CA, USA). High-quality RNAs were used to construct an Iso-Seq library. Briefly, a total of 12 PCR cycles of amplification were performed using PrimeSTAR GXL DNA Polymerase (Clontech, USA). After purification with AMPure® PB magnetic beads, the cDNA products were then subjected to the construction of SMRTbell template libraries using the SMRTbell Template Prep Kit 1.0 (Pacific Biosciences, USA). Finally, the purified SMRTbellTM templates were bound and sequenced on the Pacific Bioscience Sequel System platform. Sequencing reads were subjected to circular consensus sequences (CCSs) using SMRT Analysis Software (https://www.pacb.com/products-and-services/analytical-software/devnet/). Integrative Genomics Viewer (IGV) was used to visualize PacBio long-read sequence data.

### Single-cell RNA sequencing

Single Cell G Chip Kit (10x Genomics, 1000120), the cell suspension (300-600 living cells per microliter determined by Count Star) was loaded onto the Chromium single cell controller (10x Genomics) to generate single-cell gel beads in the emulsion according to the manufacturer's protocol. Reverse transcription was performed on a S1000TM Touch Thermal Cycler (Bio Rad) at 53°C for 45 min, followed by 85°C for 5 min, and hold at 4°C. The cDNA was generated and then amplified, and quality assessed using an Agilent 4200 (performed by CapitalBio Technology, Beijing). Single cell RNA-Seq library preparation According to the manufacture's introduction, Single-cell RNA-seq libraries were constructed using Single Cell 3' Library and Gel Bead Kit V3.1. The libraries were finally sequenced using an Illumina Novaseq 6000 sequencer with a sequencing depth of at least 100,000 reads per cell with pair-end 150 bp (sPtrEa1t5e0gy) (performed by CapitalBio Technology, Beijing).

### Statistical analyses

A t test was used to compare continuous data between two groups, followed by Bonferroni correction for multiple comparisons. The exact test used for each experiment is denoted in the figure legends, and data were expressed as the mean±SEM. All experiments were repeated a minimum of three times, and representative experiments are shown. Statistical significance was considered when *P*<0.05. All statistical analyses were performed using GraphPad Prism 9.0.

## Supplementary Material

Supplementary figures and tables.

## Figures and Tables

**Figure 11:**
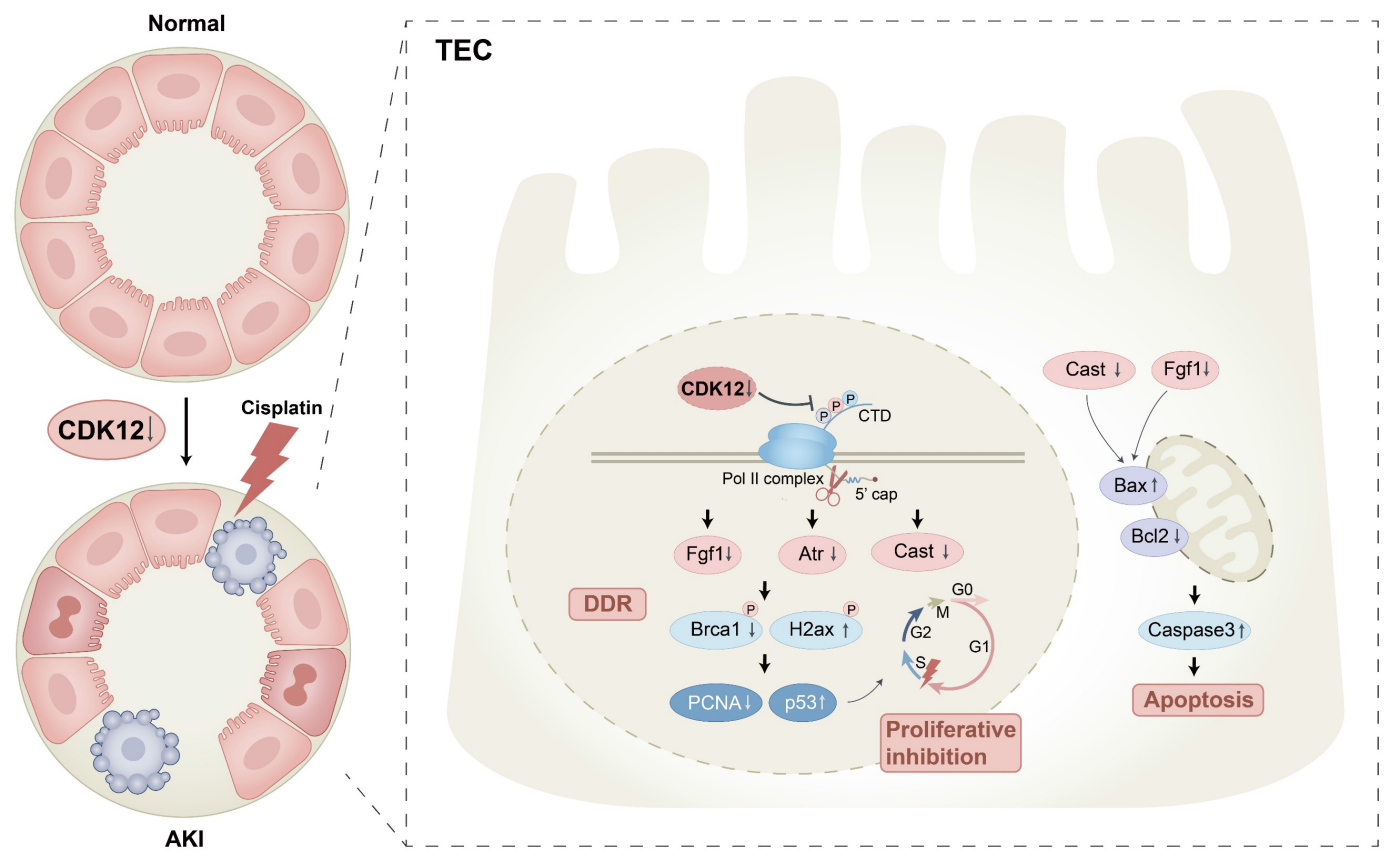
Schematic illustration of the mechanism by which CDK12 knockdown aggravates cisplatin-induced AKI. In cisplatin-induced AKI, CDK12 knockdown could lead to DNA damage, apoptosis and abrogate cell proliferation via transcriptional elongation defects in *Fgf1* and *Cast*.
