# Supplementary material for: Tubule-specific cyclin-dependent kinase 12 knockdown potentiates kidney injury through transcriptional elongation defects
Source: Int J Biol Sci. 2024 Feb 12;20(5):1669–87. doi: 10.7150/ijbs.90872 (PMC10929189; doi:10.7150/ijbs.90872)
Supplement: Supplementary file 1 — Supplementary figures and tables. [file ijbsv20p1669s1.pdf]

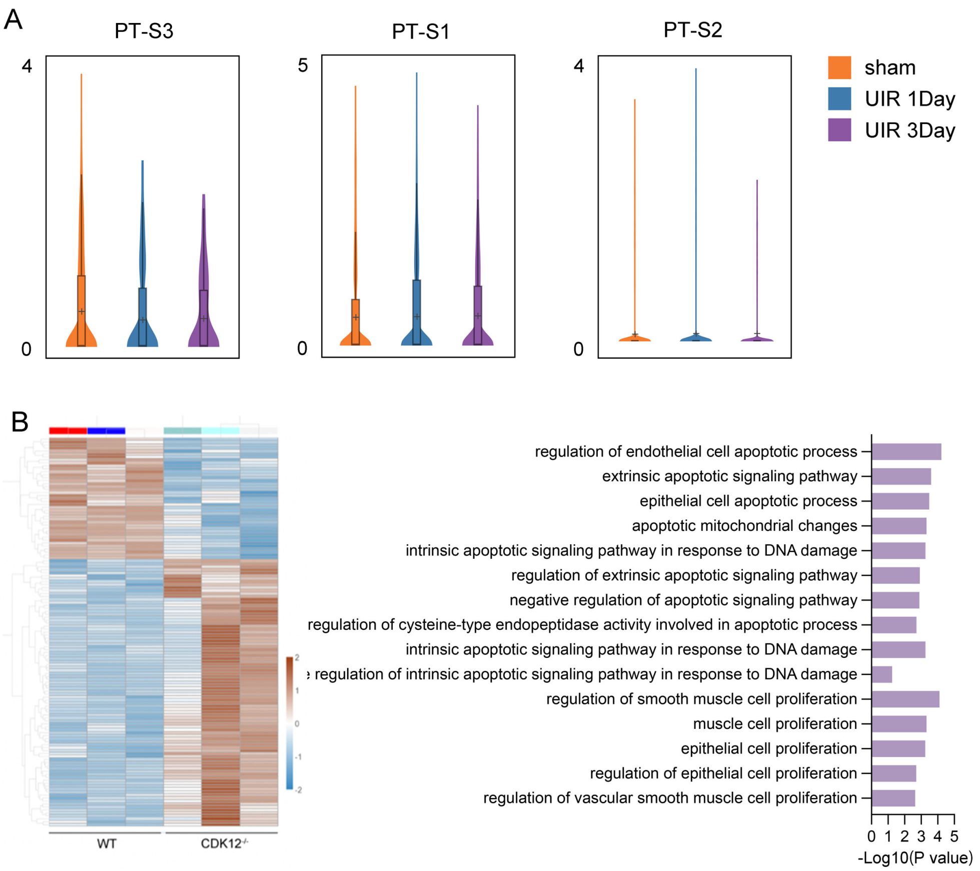

S1: RNA sequencing reveals increased apoptosis, DNA damage, and proliferation in WT and CDK12$^{RTEC-/-}$ mice. (A) Violin plots demonstrating CDK12 in UIR mice. (B) Heatmap of combined differentially expressed genes between the WT and CDK12$^{RTEC+/-}$ mice and a combination of GO enrichment analysis for differentially expressed genes.

7

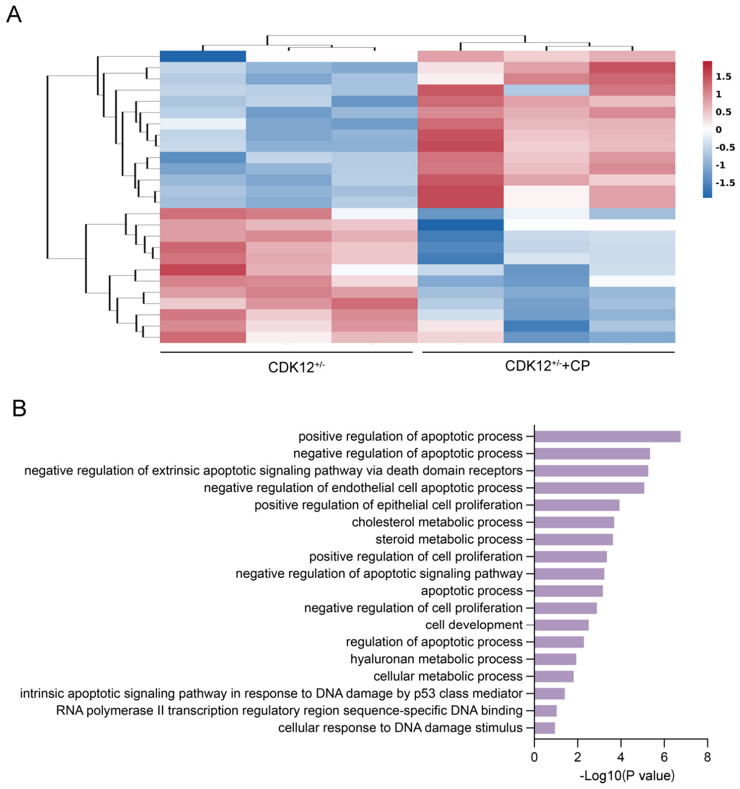

S2: RNA sequencing reveals increased apoptosis, DNA damage, and proliferation in CDK12$^{RTEC+/-}$ mice and CDK12$^{RTEC+/-}$ treated with cisplatin mice. (A) Heatmap of combined differentially expressed genes between the CDK12$^{RTEC+/-}$ mice and CDK12$^{RTEC+/-}$ treated with cisplatin mice. (B) A combination of GO enrichment analysis for differentially expressed genes.

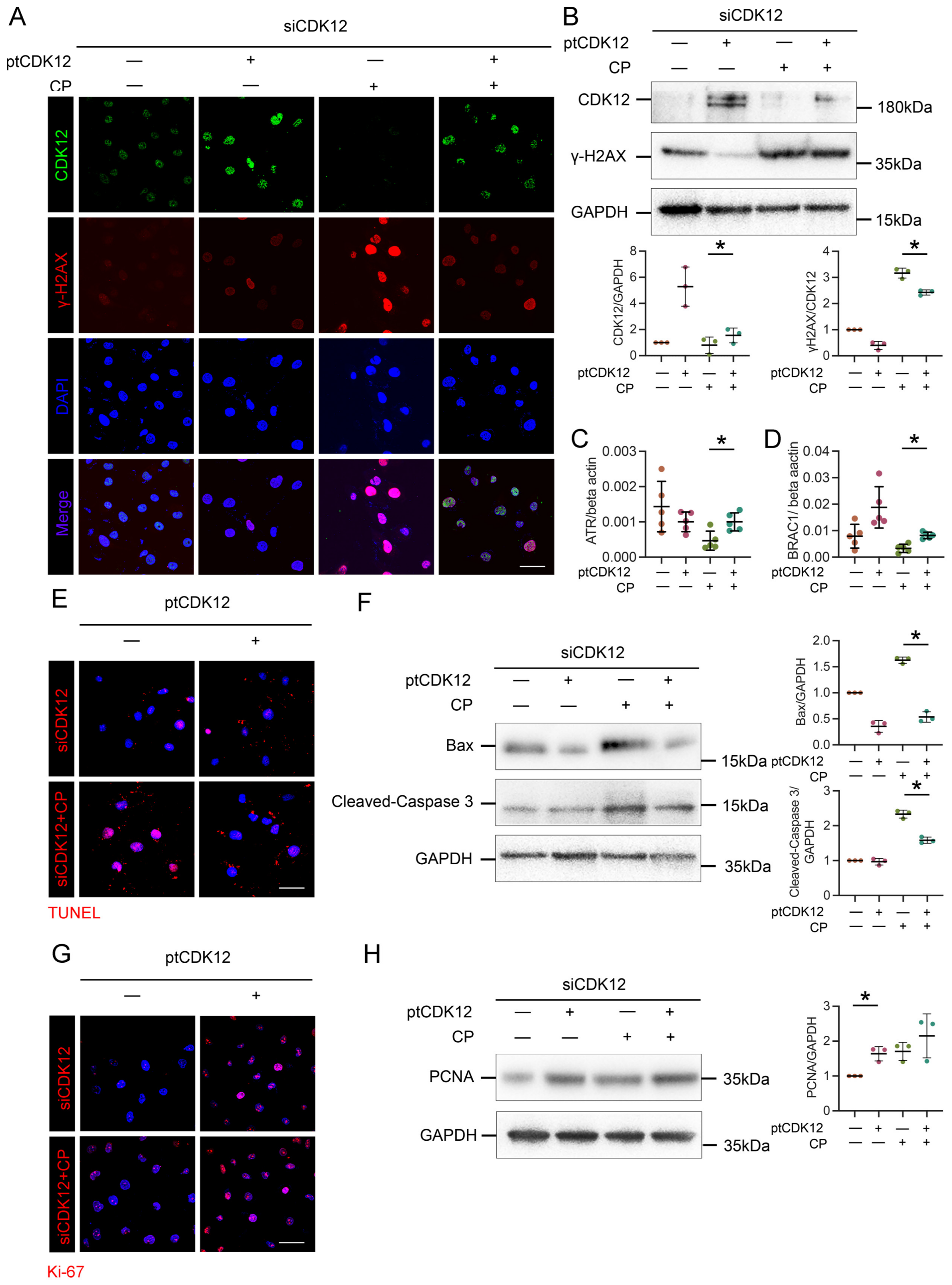

S3: Overexpression of CDK12 improves tubular injury after cisplatin treatment *in vitro*. (A) Representative immunostaining micrographs show CDK12 (green) and γ-H2AX (red) expression in four *in vitro* groups, as indicated (scale bar = 50 μm). (B) Representative western blots show that γ-H2AX expression was downregulated after treatment of CDK12 overexpression plasmid. Graphical representations of CDK12

and γ-H2AX levels in four groups, as indicated. *$P<0.05$ versus siCDK12 with cisplatin (n=5). (C) Expression of ATR and (D) BRCA1 in different groups was assessed by real-time PCR. *$P<0.05$ versus siCDK12 with cisplatin (n=5). (E) Representative immunostaining micrographs show CDK12 (green) and TUNEL (red) expression in different groups (scale bar = 50 μm). (F) Representative western blots showing the HK-2 expression of Bax and cleaved-Caspase3 in four groups. Graphical representations of Bcl-2, Bax and cleaved-Caspase3 levels in four groups, as indicated. *$P<0.05$ versus siCDK12 with cisplatin (n=5). (G) Representative immunostaining micrographs show Ki-67 expression in four groups (scale bar = 50 μm). (H) Representative western blots showing the HK-2 expression of PCNA in four groups. Graphical representations of PCNA levels in four groups, as indicated. *$P<0.05$ versus siCDK12 with cisplatin (n=5). ptCDK12, CDK12 overexpression plasmid.

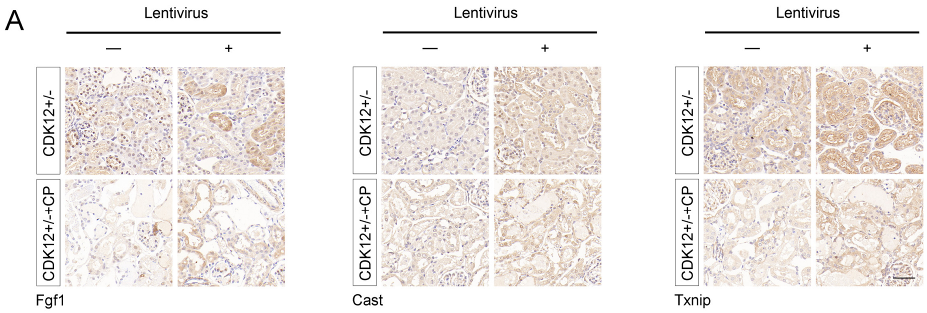

S4: Overexpression of CDK12 after cisplatin treatment largely preserved the expression of Cast, Fgf1, and Txnip in tubules. A. Representative immunostaining micrographs show Txnip, Cast, and Fgf1 expression in different groups, as indicated. (scale bar = 50 μm).

Supplemental Table 1. Clinical characteristics of all patients

Number	Gender	Pathology	Age	Scr(umol/L)	BUN(mmol/L)	Tubular injury score
1	Female	Acute tubular necrosis	69	319.2	19.44	4
2	Female	Acute tubular necrosis	62	217	17.21	3
3	Male	Acute tubular necrosis	60	964	23.77	4
4	Male	Acute tubular injury	66	124	6.3	2
5	Male	Acute tubular injury	41	65	8.91	1
6	Male	Acute tubular injury	53	114	7.3	2
7	Female	Acute tubular injury, minor glomerular abnormalities	62	112	5.58	1
8	Male	Acute tubular necrosis	67	229	9.9	3
9	Male	Acute tubular injury	43	242	17.6	3
10	Male	Minimal change disease, acute tubular injury	19	86.9	6.83	1
11	Male	Minimal change disease, acute tubular injury	36	129	11.2	2
12	Male	Acute tubular necrosis	54	216	15.09	3
13	Male	Acute tubular injury	48	120.2	12.76	2
14	Female	Minimal change disease, acute tubular injury	62	97	5.25	2
15	Female	Acute tubular necrosis	41	601	22.26	4
16	Male	Acute tubular necrosis	55	785.5	17.55	4
17	Male	Acute tubular necrosis	35	1034	31.7	4
18	Male	Acute tubular injury	40	197	13.4	3
19	Male	Acute tubular injury	30	125	8.3	2
20	Male	Acute tubular injury	23	111	5.18	1

Supplemental Table 2. Primers used in this study

Primers for quantitative Real Time-PCR<br>S: sense primer<br>A: antisense primer	
M-Med16(1)-S	TGGGTGCGGTGGGTATGAT
M-Med16(1)-A	CAGATCCTGGTCATCATTGCG
M-Med16(2)-S	TAACCATGGGAAGCTCAGCAT
M-Med16(2)-A	ACGTGTAGCAGGATGTCCCAC
M-Med16(3)-S	TTCACAGTACATAAAGCCCCTTGT
M-Med16(3)-A	AACTTTATTGGGCCAGTCTTCC
M-Smug1(1)-S	GGACTTGCAAAGCAAAGGTCC
M-Smug1(1)-A	ATTTTGGCTCCAACCAGTGG
M-Smug1(2)-S	CTACCCATGAGCCTGCAAGC
M-Smug1(2)-A	GCTCCCAAGCATAATCCACC
M-Smug1(3)-S	TTCCCCAGTCAGTGGACAATCT
M-Smug1(3)-A	CAAGCTGGCCTTGAACTCAATAT
M-Txnip(1)-S	GCTGAAACTTCCAGGCACCTT
M-Txnip(1)-A	AGCTCGCCTCCGTAAAGTCAG
M-Txnip(2)-S	AATATGAGTACAAGTTCGGCTTCG
M-Txnip(2)-A	GCAGACACTGGTGCCATTAGGT
M-Txnip(3)-S	TGCTGACCTTCTGGCGTTGT
M-Txnip(3)-A	CCATGACTTGAAATTGGCTCTG
M-Cast(1)-S	AGCTCTGGGTTGCTGAGAAGTT
M-Cast(1)-A	GGAAAACGCAGCGAAATTGT
M-Cast(2)-S	CTGCCTTGGATGACCTGATAGA

M-Cast(2)-A	CCAGTGCCTCAAGGTAGGTAGA
M-Cast(3)-S	ATCTCCTTTCCTGCCTGAACTC
M-Cast(3)-A	ATGTAGAGGGTGGAAACCACAGT
M-Fgf1(1)-S	AGCTGCAGAAATCCTGAGGC
M-Fgf1(1)-A	CTCAGCACTGAAGAACTGGCA
M-Fgf1(2)-S	GATGGGACAAGGGACAGGAG
M-Fgf1(2)-A	CTCATTTGGTGTCTGCGAGC
M-Fgf1(3)-S	CGGAACAGAGTAAGAAGGCAAGA
M-Fgf1(3)-A	TCCTGTCTCCTTGCTCCTACG
ATR-S	CTCTGCATACGTCCTACCATTG
ATR-A	GACTGCTGTACTGGGTGTTATG
BRCA1-S	CTTCTATCAGGTGTGCTCTTCC
BRCA1-A	TCTTCACTGCTACCACAACTATC

44
